# How to Design AI for Social Good: Seven Essential Factors

**DOI:** 10.1007/s11948-020-00213-5

**Published:** 2020-04-03

**Authors:** Luciano Floridi, Josh Cowls, Thomas C. King, Mariarosaria Taddeo

**Affiliations:** 1 Digital Ethics Lab, Oxford Internet Institute, University of Oxford, Oxford, UK; 2 The Alan Turing Institute, London, UK

**Keywords:** AI4SG, Artificial intelligence, Ethics, Social good, Transparency, Privacy, Safety

## Abstract

The idea of artificial intelligence for social good (henceforth AI4SG) is gaining traction within information societies in general and the AI community in particular. It has the potential to tackle social problems through the development of AI-based solutions. Yet, to date, there is only limited understanding of what makes AI socially good in theory, what counts as AI4SG in practice, and how to reproduce its initial successes in terms of policies. This article addresses this gap by identifying seven ethical factors that are essential for future AI4SG initiatives. The analysis is supported by 27 case examples of AI4SG projects. Some of these factors are almost entirely novel to AI, while the significance of other factors is heightened by the use of AI. From each of these factors, corresponding best practices are formulated which, subject to context and balance, may serve as preliminary guidelines to ensure that well-designed AI is more likely to serve the social good.

## Introduction

The idea of “Artificial Intelligence (AI) for Social Good” (henceforth AI4SG) is becoming popular in many information societies and gaining traction within the AI community (Hager et al. 2017). Projects seeking to use AI for social good vary significantly. They range from models to predict septic shock (Henry et al. 2015) to game-theoretic models to prevent poaching (Fang et al. 2016); from online reinforcement learning to target HIV-education at homeless youths (Yadav et al. 2016a, b) to probabilistic models to prevent harmful policing (Carton et al. 2016) and support student retention (Lakkaraju et al. 2015). Indeed, new applications of AI4SG appear almost daily, making possible socially good outcomes that were once less easily achievable, unfeasible, or unaffordable.

Several frameworks for the design, development, and deployment of ethical AI in general have recently emerged (see Floridi et al. 2018 for a comparative analysis and synthesis). However, there is still only limited understanding about what constitutes AI “for the social good” (Taddeo and Floridi 2018a). Approaching AI4SG ad hoc, by analysing specific areas of application—like famine-relief or disaster management—as an annual summit for AI industry and government has done (ITU 2017, 2018; “AI for Good Global Summit” 2019) indicates the presence of a phenomenon, but neither explains it, nor does it suggest how other AI4SG solutions could and should be designed to harness AI’s full potential. Furthermore, many projects that generate socially good outcomes using AI are not (self-)described as such (Moore 2019).

Lacking a clear understanding of what makes AI socially good in theory, what may be described as AI4SG in practice, and how to reproduce its initial successes in terms of policies is a problem because designers of AI4SG face at least two main challenges: unnecessary failures and missed opportunities. AI software is shaped by human values which, if not carefully selected, may lead to “good-AI-gone-bad” scenarios. For example, consider the failure of IBM’s oncology-support software, which attempts to use machine learning to identify cancerous tumours, but which was rejected by medical practitioners “on the ground” (Ross and Swetlitz 2017). The system was trained using synthetic data and was not sufficiently refined to interpret ambiguous, nuanced, or otherwise “messy” patient health records (Strickland 2019). It also relied on US medical protocols, which are not applicable worldwide. The heedless deployment and the poor design of the software led to misdiagnoses and erroneous treatment suggestions, breaching the trust of doctors. Context-specific design and deployment could help prevent such value misalignment and deliver successful AI4SG projects on a more consistent basis.

At the same time, the genuinely socially good outcomes of AI may arise merely by chance, for example through an accidental application of an AI solution in a different context. This was the case with the use of a different version of IBM’s cognitive system. In this case, the Watson system was originally designed to identify biological mechanisms, but when used in a classroom setting, it inspired engineering students to solve design problems (Goel et al. 2015). In this instance, AI provided a unique mode of education. But lacking a clear understanding of AI4SG means that this success is accidental and cannot be repeated systematically, whilst for each “accidental success” there may be countless examples of missed opportunities to exploit the benefits of AI for advancing socially good outcomes in different settings.

In order to avoid unnecessary failures and missed opportunities, AI4SG would benefit from an analysis of the essential factors that support and underwrite the design and deployment of successful AI4SG. In this article, we provide the first, fine-grained analysis of these factors. Our aim here is not to document every single ethical consideration for an AI4SG project. For example, it is essential, and hopefully self-evident, that an AI4SG project ought not to advance the proliferation of weapons of mass destruction, an imperative which we do not discuss here (Taddeo and Floridi 2018b). Likewise, it is important to acknowledge at the outset that there are myriad circumstances in which AI will not be the most effective way to address a particular social problem. This could be due to the existence of alternative approaches that are more efficacious (i.e., “Not AI For Social Good”) or because of the unacceptable risks that the deployment of AI would introduce (i.e., “AI For Insufficient Social Good” as weighed against its risks). Nor do we foresee many (or perhaps any) cases in which AI is a “silver bullet”—the single-handed solution to an entrenched social problem (i.e., “Only AI for Social Good”). What is therefore essential about the factors and the corresponding best practices is not their incorporation in every circumstance; we note several examples where it would be morally defensible not to incorporate a particular factor. Instead, what is essential is that each best practice is (i) considered proactively, and (ii) not incorporated if and only if there is a clear, demonstrable, and morally defensible reason why it should not be.

In this article, we focus on identifying factors that are particularly relevant to AI as a technological infrastructure, to the extent that it is designed and used for the advancement of social good. To anticipate, these seven factors are: (1) falsifiability and incremental deployment; (2) safeguards against the manipulation of predictors; (3) receiver-contextualised intervention; (4) receiver-contextualised explanation and transparent purposes; (5) privacy protection and data subject consent; (6) situational fairness; and (7) human-friendly semanticisation. With these factors identified, the questions that are likely to arise in turn are: how these factors ought to be evaluated and resolved, by whom, and with what supporting mechanism (e.g. regulation or codes of conduct). These questions, which are not within the scope of this article and will be addressed in the next stage of this research, are intertwined with wider ethical and political issues regarding the legitimacy of decision-making with, and about, AI.

The rest of the article is structured as follows. In section two, we explain how we identified the seven factors. In section three, we analyse the seven factors individually. We elucidate each of them by reference to one or more case studies, and we derive from each factor a corresponding best practice for AI4SG creators to follow. In the concluding section, we discuss the factors and suggest how tensions between them may be resolved.

## Methodology

AI4SG initiatives are successful insofar as they help to reduce, mitigate or eradicate a given problem of moral significance. Thus, our analysis of the essential factors for successful AI4SG is based on the following working definition:

AI4SG =_def._ the design, development, and deployment of AI systems in ways that (i) prevent, mitigate or resolve problems adversely affecting human life and/or the wellbeing of the natural world, and/or (ii) enable socially preferable and/or environmentally sustainable developments.^1^

Following this definition, we analysed a set of 27 projects, obtained via a systematic review of relevant literature undertaken by the authors, to identify clear and significant cases of successful and unsuccessful examples of AI4SG. The literature analysis that underpins this article involved searching five databases (Google Scholar, PhilPapers, Scopus, SSRN, and Web of Science), between October 2018 and May 2019. We initially conducted a broad search for AI for Social Good on each of these search engines. This general search returned many results on AI’s application for good. Hence, we searched for uses of AI in areas related to human life and the wellbeing of the natural world, like ‘healthcare’, ‘education’, ‘equality’, ‘climate change’, and ‘environmental protection’. This provided disjoined keywords from which we derived chosen synonyms to perform area-specific searches. Each research-area search used the query: < area and synonyms > AND ("Artificial Intelligence" OR "Machine Learning" OR "AI ") AND “Social Good”. From the set of 27 cases, we identified 7 cases (see Appendix for a list) as being most representative in terms of scope, variety, impact, and for their potentiality to corroborate the essential factors that we argue should characterise the design of AI4SG projects.

The factors described in this article have been identified in coherence with more general work in the field of AI ethics. Each factor relates to at least one of five ethical principles of AI—*beneficence*, *nonmaleficence*, *justice*, *autonomy*, and *explicability*—identified in the comparative analysis mentioned above (Floridi et al. 2018). This coherence is crucial: AI4SG cannot be inconsistent with the ethical framework guiding the design and evaluation of AI in general. The principle of beneficence is of particular relevance. It states that the use of AI should provide benefit to people and the natural world, and indeed AI4SG projects should not just comply with but *reify* this principle, such that the benefits of AI4SG should be preferable and sustainable, in line with the definition above. Beneficence is thus a necessary condition of AI4SG, yet it is insufficient, not least because the beneficent impact of an AI4SG project may be “offset” by the creation or amplification of other risks or harms.^2^ Moreover, while others of these ethical principles, such as autonomy and explicability, indeed recur throughout our discussion, the factors we evince below are more closely associated with design considerations that are specific to AI4SG, and may be operationalised in the form of the corresponding best practices provided for each. In this way, ethical analysis informing the design and the deployment of AI4SG initiatives has a central role in mitigating foreseeable risks of unintended consequences and possible misuses of the technology.

Before discussing the factors, it is important to clarify three general features of the whole set: *dependency*, *order*, and *coherence*. The seven factors are often intertwined and co-dependent, but for the sake of simplicity we discuss them separately. Nothing should be inferred from this choice. In the same way, the factors are all essential, none of them is “more important” than any other, so we shall introduce them not in terms of priority, but somewhat historically, starting with factors that pre-date AI, and yet take on greater importance when AI technologies are used, owing to the particular capabilities and risks of AI (Yang et al. 2018).^3^ These include *falsifiability and incremental deployment* and *safeguards against the manipulation of data*. There are also factors that relate more intrinsically to the sociotechnical characteristics of AI as it exists today, like *situational fairness* and *human-friendly semanticisation*.

The factors are ethically robust and pragmatically applicable, in the sense that they give rise to design considerations in the form of best practices that should be ethically endorsed. It is crucial to stress here that the seven factors we identify are not by themselves *sufficient* for socially good AI, but careful consideration of each of them is, we argue, necessary. Hence, the set of factors we identify should not be taken as a “checklist” which, if merely complied with, guarantees socially good outcomes from the use of AI in a particular domain. In the same vein, we highlight the need to strike a balance between the different factors, and indeed between tensions that may arise even within a single factor. It follows that seeking to frame a project as “for social good” or “not for social good” in a binary way seems needlessly reductive, not to mention subjective. The aim of the article is not to identify, or offer the means to identify AI4SG projects; our goal is to identify ethically important characteristics of projects that could feasibly be described as AI4SG.

## Seven Essential Factors for Successful AI4SG

As we anticipated, the factors are (1) *falsifiability and incremental deployment*; (2) *safeguards against the manipulation of predictors*; (3) *receiver-contextualised intervention*; (4) *receiver-contextualised explanation and transparent purposes*; (5) *privacy protection and data subject consent*; (6) *situational fairness*; and (7) *human-friendly semanticisation*. We shall elucidate each factor with one or more examples of projects in the sample, and offer a corresponding best practice.

### Falsifiability and Incremental Deployment

Trustworthiness is essential for technology in general (Taddeo and Floridi 2011; Taddeo 2017), and for AI4SG applications in particular, to be adopted and have a meaningful positive impact on human life and environmental wellbeing. Trustworthiness of an AI application entails a high probability that the application will respect the principle of beneficence, or at the very least the principle of nonmaleficience. While there is no universal rule or guideline that can ensure or guarantee trustworthiness, *falsifiability* is an essential factor to improve the trustworthiness of technological applications in general, and AI4SG applications in particular.

Falsifiability entails the specification, and the possibility of empirical testing, of one or more critical requirements, that is, an essential condition, resource, or means for a capability to be fully operational, such that something could or should not work without it. *Safety* is an obvious critical requirement. Hence, for an AI4SG system to be trustworthy, its safety should be falsifiable.^4^ If falsifiability is not possible, then the critical requirements cannot be checked, and then the system should not be deemed trustworthy. This is why falsifiability is an essential factor for all conceivable AI4SG projects.

Unfortunately, we cannot know for sure that a given AI4SG application is safe unless we can test the application in all possible contexts. In this case, the map of testing would simply equate to the territory of deployment. As this *reductio ad absurdum* makes clear, complete certainty is out of reach. What is within reach, in an uncertain and fuzzy world with many unforeseen situations, is the possibility to know when a given critical requirement is not implemented or may be failing to work properly. Hence, if the critical requirements are falsifiable, we can know when the AI4SG application is not trustworthy, but not whether it is trustworthy.

Critical requirements should be tested with an incremental deployment cycle. Unintended hazardous effects may only reveal themselves after testing. At the same time, software should only be tested in the real world if it is safe to do so. This requires adoption of a deployment cycle whereby developers: (a) ensure that the application’s most critical requirements or assumptions are falsifiable, (b) undertake hypothesis testing of those most critical requirements and assumptions in safe, protected contexts, and, if these hypotheses are not disproven over a small set of suitable contexts, then (c) conduct testing across increasingly wide contexts, and/or test a larger set of less critical requirements, and all this while (d) being ready to halt or modify the deployment as soon as hazardous or other unwanted effects may appear.

AI4SG applications may use formal approaches to try to test critical requirements. For example, they may include the use of formal verification to ensure that autonomous vehicles, and AI systems in other safety–critical contexts, would make the ethically preferable choice (Dennis et al. 2016). Such methods offer safety checks that, in terms of falsifiability, can be proved correct. Simulations may offer roughly similar guarantees. A simulation enables one to test whether critical requirements (again, consider safety) are met under a set of formal assumptions. Unlike a formal proof, a simulation cannot always indicate that the required properties are necessarily always satisfied. But a simulation often enables one to test a much wider set of cases that cannot be dealt with formally, e.g., due to the complexity of the proof.

It would be misguided to rely purely on formal properties or simulations to falsify an AI4SG application. The assumptions of these models cage the real-world applicability of any conclusions that one might make. And assumptions may be incorrect in reality. What one may prove to be correct via a formal proof, or likely correct via testing in simulation, may be disproved later with the real-world deployment of the system. For example, developers of a game-theoretic model for wildlife security assumed a relatively flat topography without serious obstructions. Hence, the software that they developed originally had an incorrect definition of an optimal patrol route. Incremental testing of the application enabled the refinement of the optimal patrol route by proving wrong the assumption of a flat topography (Fang et al. 2016).

If novel dilemmas in real-world contexts require the alteration of prior assumptions made in the lab, one solution is to rectify a priori assumptions after deployment. Alternatively, one may adopt an “on-the-fly” or runtime system for a constant update of a program’s processing (“understanding”) of its inputs. Yet, problems also abound with this approach. For example, Microsoft’s infamous Twitter bot, Tay, acquired meanings, in a very loose sense, at runtime, as it learned from Twitter users how it should respond to tweets. After deployment in the real—and frequently vicious—world of social media, however, the bot’s ability to adapt constantly its “conceptual understanding” became an unfortunate bug, as Tay “learned” and regurgitated offensive language and unethical associations between concepts from other users (Neff and Nagy 2016).

The use of a retrodictive approach—that is, an attempt to understand some aspect of reality through a priori information—to deal with the falsifiability of requirements presents similar problems. This is noteworthy, since retrodiction is the primary method of supervised machine learning approaches that learn from data (e.g., the learning of a continuous transformation function in the case of neural networks).

From the previous analysis it follows that the essential factor of falsifiability and incremental development comprises a cycle: engineering requirements that are falsifiable (so that it is at least possible to know whether the requirements are not met); falsification testing for incrementally improving levels of trustworthiness; adjustment of a priori assumptions; and then and only then deployment in an incrementally wider and critical context. Germany’s approach to regulating autonomous vehicles offer a good example of this incremental approach. Deregulated zones allow experimentation of constrained autonomy and, after increasing the levels of trustworthiness, manufacturers may test vehicles with higher levels of autonomy (Pagallo 2017). Indeed, the creation of such deregulated zones, or *teststrecken,* was one recommendation to support more ethical AI policy at the European level (Floridi et al. 2018). The identification of this essential factor yields the following best practice:

1. AI4SG designers should identify falsifiable requirements and test them in incremental steps from the lab to the “outside world”.

### Safeguards Against the Manipulation of Predictors

The use of AI to predict future trends or patterns is very popular in AI4SG contexts, from applying automated prediction to redress academic failure (Lakkaraju et al. 2015), to preventing illegal policing (Carton et al. 2016), and detecting corporate fraud (Zhou and Kapoor 2011). The predictive power of AI4SG faces two risks: the manipulation of input data, and excessive reliance on non-causal indicators.

The manipulation of data is not a new problem, nor is it limited to AI systems alone. Well-established findings such as Goodhart’s Law (Goodhart 1975), which is often summarised as “when a measure becomes a target, it ceases to be a good measure” (Strathern 1997, 308), long pre-date widespread adoption of AI systems. But in the case of AI, the problem of data manipulation may be exacerbated (Manheim and Garrabrant 2019) and lead to unfair outcomes that breach the principle of justice. As such, it is a noteworthy risk for any AI4SG initiative, because it can impair the predictive power of AI and lead to the avoidance of socially good interventions at the individual level. Consider the concern raised by Ghani over teachers who face being evaluated in respect to:

the percentage of students in their class who are above a certain risk threshold. If the model was transparent — for example, heavily reliant on math GPA — the teacher could inflate math grades and reduce the intermediate risk scores of their students (Ghani 2016).

As Ghani goes on to argue, the same concern applies to predictors of adverse police officer interactions:

these systems [are] very easy to understand and interpret, but that also makes them easy to game. An officer who has had two uses of force in the past 80 days may choose to be a bit more careful over the next 10 days, until the count rolls over to zero again.

These hypothetical examples make clear that, when the model used is an easy one to understand “on the ground”, it is already open to abuse or “gaming”, independently of whether AI is used. The introduction of AI complicates matters, owing to the scale at which AI is typically applied.^5^As we have seen, if the information used to predict a given outcome is known, an agent with such information (that is predicted to take a particular action) can change each predictive variable’s value in order to avoid an intervention. In this way, the predictive power of the overall model is reduced, as it has been shown by empirical research in the domain of corporate fraud (Zhou and Kapoor 2011). Such a phenomenon could carry across from fraud detection to the domains that AI4SG initiatives seek to address.^6^

At the same time, there is a risk that excessive reliance on non-causal indicators—that is, data which is correlated with, but not causal of, a phenomenon—may distract attention from the context in which the AI4SG designer is seeking to intervene. To be effective, any such intervention should alter the underlying causes of a given problem, such as a student’s domestic problems or inadequate corporate governance, rather than non-causal predictors. To do otherwise is to risk addressing only a symptom, rather than the root cause of a problem.

These risks suggest the need to consider the use of safeguards as a design factor for AI4SG projects. Such safeguards may constrain the selection of indicators to be used in the design of AI4SG projects; the extent to which these indicators should shape interventions; and/or the level of transparency that should apply to how indicators affect decision. This yields the following best practice:

(2) AI4SG designers should adopt safeguards which (i) ensure that non-causal indicators do not inappropriately skew interventions, and (ii) limit, when appropriate, knowledge of how inputs affect outputs from AI4SG systems, to prevent manipulation.

### Receiver-Contextualised Intervention

It is essential that software intervenes in users’ life only in ways that respect their autonomy. Again, this is not a problem that arises only with AI-driven interventions, but the use of AI introduces new considerations. In particular, a core challenge for AI4SG projects is to devise interventions that balance current and future benefits. The balancing problem, which is familiar to preference-elicitation research (Boutilier 2002; Faltings et al. 2004; Chajewska et al. 2000), boils down to a temporal choice interdependency. An intervention in the present can elicit user preferences that then enable the software to contextualise future interventions to the given user. Consequently, an intervention strategy that has no impact on user autonomy (e.g., one that lacks any interventions) may be ineffective in extracting the necessary information for correctly contextualised future interventions. Conversely, an intervention that overly infringes upon a user’s autonomy may cause the user to reject the technology, making future interventions impossible.

This balancing consideration is a common one for AI4SG initiatives. Take, for example, interactive activity recognition software for people with cognitive disabilities (Chu et al. 2012). The software is designed to prompt patients to maintain a daily schedule of activities (e.g., taking medication), whilst minimising interruptions to their wider goals. Each intervention is contextualised in such a way that the software learns the timing of future interventions from responses to past interventions. Moreover, only important interventions are made, and yet all interventions are partially optional because declining one prompt leads to the same prompt later on. Here, the concern was that patients would reject an overly intrusive technology; hence a balance was sought. This balance is lacking in our second example. A game-theoretic application intervenes in wildlife security officers’ patrols by offering suggested routes (Fang et al. 2016). If a route poses physical obstacles, however, then the software lacks the possibility to provide alternative suggestions. Officers may ignore the advice by taking a different route, but not without disengaging from the application. It is essential to relax such constraints, so that users can ignore an intervention, but accept subsequent, more appropriate interventions (in the form of advice) later on.

These examples point to the importance of seeing users as equal partners in both the design and deployment of autonomous decision-making systems. The adoption of this mindset might have helped prevent the tragic loss of two Boeing 737 Max airliners. It appears that the pilots of these flights struggled to reverse a software malfunction caused by faulty sensors, due in part to the absence of “optional safety features” which Boeing sold separately (Tabuchi and Gelles 2019).

The risk of false positives (unnecessary intervention, creating disillusionment) is often just as problematic as false negatives (no intervention where it is necessary, limiting effectiveness). Hence, a suitable receiver-contextualised intervention is one that achieves the right level of disruption while respecting autonomy through optionality. This contextualisation rests on information about users’ capacities, preferences and goals, and the circumstances in which the intervention will take effect.

One can consider five dimensions relevant to a receiver-contextualised intervention. Four of these dimensions emerge from McFarlane’s taxonomy of interdisciplinary research on disruptive computer–human interruptions (McFarlane 1999; McFarlane and Latorella 2002 17–19). These are: the individual characteristics of the person receiving the intervention; the methods of coordination between the receiver and the system; the meaning or purpose of the intervention; and the overall effects of the intervention.^7^ A fifth dimension of relevance is optionality: a user can choose either to ignore all offered advice or to drive the process and request a different intervention better suited to their needs.

We can summarise these five dimensions in the form of the following best practice for receiver-contextualised intervention.

(3) AI4SG designers should build decision-making systems in consultation with users interacting with, and impacted, by these systems; with understanding of users’ characteristics, of the methods of coordination, and the purposes and effects of an intervention; and with respect for users’ right to ignore or modify interventions.

### Receiver-Contextualised Explanation and Transparent Purposes

AI4SG applications should be designed to make explainable the operations and outcomes of these systems and to make transparent their purposes. These two requirements are of course intrinsically linked, as the operations and outcomes of AI systems reflect the wider purposes of human designers; in this section, we address both in turn.

Making AI systems explainable is an important ethical principle (Floridi et al. 2018). It has been a focus of research since at least 1975 (Shortliffe and Buchanan 1975). And it has gained more attention recently (Thelisson et al. 2017; Wachter et al. 2016) given the increasingly pervasive distribution of AI systems. As we saw above, AI4SG projects should offer interventions that are contextualised to the receiver. In addition, the *explanation* for an intervention should be contextualised in order to be adequate, and protect the autonomy of the receiver.

Designers of AI4SG projects have tried to increase the explainability of decision-making systems in various ways. For example, researchers have used machine learning to predict academic adversity (Lakkaraju et al. 2015). These predictors used concepts that the school officials interpreting the system found familiar and salient, such as GPA scores and socio-economic categorisations. Researchers have also used reinforcement-learning to help officials at homeless shelters educate homeless youths about HIV (Yadav et al. 2016a, b). The system learns how to maximise the influence of HIV education, by choosing which homeless youths to educate, on the basis that homeless youths may pass on their knowledge. One version of the system explained which youth was chosen by revealing their social network graph. However, the homeless shelter officials found that these explanations were counter-intuitive, potentially affecting the understanding of how the system worked and, hence, users’ trust in the system. These two cases exemplify the importance of the right conceptualisation when explaining an AI-based decision.

The right conceptualisation is likely to vary between AI4SG projects, because they differ greatly in their objectives, subject matter, context and stakeholders. The conceptual framework, that is, the Level of Abstraction (LoA) depends on what is being explained and to whom (Floridi 2017). An LoA is a key component of a theory, and hence of any explanation. A theory comprises five components:

1. a *System*, which is the referent or object analysed by a theory;
2. a *Purpose*, which is the “what for” that motivates the analysis of a system (note that this answers the question “what is the analysis for?” and should not be confused with a system’s purpose, which answers the question “what is the system for?”. Below, we use the term “goal” for system’s purpose whenever there may be a risk of confusion);
3. a *Level of Abstraction*, which provides a lens through which a system is analysed, and generates;
4. a *Model*, that is, some relevant and reliable information about the analysed system, which identifies;
5. a *Structure* of the system, which comprises the features that belong to the system being analysed.

There is an interdependency between the choice of the specific purpose, the relevant LoA that can fulfil the purpose, the system analysed, and the model obtained by analysing the system at a specified LoA for a particular purpose. The LoA provides the conceptualisation of the system (e.g., GPA scores, and socio-economic backgrounds). But the purpose constrains the construction of LoAs. For example, if we choose to explain the decision making system itself (e.g., the use of particular machine learning techniques), then the LoA can only conceptualise those AI techniques. In turn, the LoA generates the model, which explains the system. The model identifies system structures, such as a specific student’s GPA score, poor attendance rate, and their socioeconomic background being predictors of their academic failure. Consequently, designers must choose carefully the purpose and the corresponding LoA, so that the explanation model can provide the right explanation of the system in question for a given receiver.

A LoA is chosen for a specific *purpose*: for example, a LoA chosen to explain a decision taken on the basis of outcomes obtained through an algorithmic procedure varies depending on whether the explanation is meant for the receiver of that decision or for an engineer responsible for the design of the algorithmic procedure. This is because, depending on the purpose and its granularity (e.g. a customer-friendly vs. engineer-friendly explanation), not every LoA is appropriate for a given receiver. Sometimes, a receiver’s conceptual view of the world may differ from the one on which the explanation is based. In other cases, a receiver and an explanation may be conceptually aligned, but the receiver may not agree on the amount of granularity (LoA) of the information (what we called more precisely the model) provided. Conceptual disalignment means that the receiver may find the explanation irrelevant, unintelligible or, as we shall see below, questionable. In respect of (un)intelligibility, a LoA may use unknown labels (so-called observables), or labels that have different meanings for different users.

Empirical studies (Gregor and Benbasat 1999) suggest that the suitability of an explanation differs among receivers according to their expertise. Receivers may require explanations about how the AI software came to a decision, especially when they must take action based on that decision (Gregor and Benbasat 1999; Watson et al. 2019). How the AI system came to a conclusion can be just as important as the justification for that conclusion. Consequently, designers must also contextualise the method of explanation to the receiver.

The case of the software that uses influence-maximisation algorithms to target homeless youths for HIV education provides a good example of the relevance of the receiver-contextualisation of concepts (Yadav et al. 2016a, b). The researchers involved in this project considered three possible LoAs when designing the explanation model: the first LoA included utility calculations; the second LoA focused on social graph connectivity; and a third LoA focusing on pedagogic purpose. The first LoA highlighted the utility of targeting one homeless youth over another. According to the researchers, in this case, homeless shelter workers (the receivers) might have misunderstood the utility calculations or found them irrelevant. Utility calculations offer little explanatory power beyond the decision itself, because they often simply show that the “best” choice was made, and how good it was. Explanations based on the second LoA faced a different problem: the receivers assumed that the most central nodes in the network were the best for maximising the influence of education, while the optimal choice is often a set of less well-connected nodes. This disjuncture may have arisen from the nature of the connectivity between members of the network of homeless youths, which reflects real-life uncertainty about friendships. Since who counts as a “friend” is often vague and changeable over time, the researchers classified edges in the network as either “certain” or “uncertain” based on domain knowledge. For “uncertain” relationships, the probability of a friendship existing between two youths was determined by domain experts.^8^ The third LoA was eventually chosen, after subsequent user testing of different explanation frameworks (Yadav et al. 2016a, b). In light of their stated goal to justify decisions in a way that would be intuitive to homeless shelter officials, the researchers considered omitting references to the Maximum Expected Utility (MEU) calculations, even though this is what actually underlies the decisions made by the system. Instead, the researchers considered justifying decisions using concepts with which officials would be more comfortable and familiar, such as the centrality of the nodes (i.e., the youths) that the system recommends officials prioritise for intervention. In this way, the researchers sought to provide the most relevant information contextualised to the receiver.

As this example shows, given a particular system, the purpose one chooses to pursue when seeking an explanation of it, at what LoA, and the issuing model that is obtained are crucial variables that impact the effectiveness of an explanation. Explainability breeds trust in, and fosters adoption of, AI4SG solutions (Herlocker et al. 2000; Swearingen and Sinha 2002; Bilgic and Mooney 2005). This is why it is essential that software uses persuasive *argumentation* for the target audience. This is likely to include information about both the general functionality and logic employed by a system and the reasons for the specific decision being made (Wachter et al. 2017).

Transparency in the goal (i.e., system’s purpose) of the system is also crucial, for it follows directly from the principle of autonomy. Consider, for example, the development of AI solutions to prompt people with cognitive disabilities to take their medication (Chu et al. 2012). On its face, this application may seem invasive, involving vulnerable users, limiting the effectiveness of receiver-conceptualised explanation. However, the system is not designed to coerce the patients into a given behaviour, nor is it designed to resemble a human being. The patients have autonomy not to interact with the AI system in question. This case highlights the importance of transparency in goals, particularly in contexts in which explainable operations and outcomes are unworkable or undesirable. Transparency in goals, thus, undergirds other safeguards around the protection of target populations and may help ensure compliance with relevant legislation and precedent (Reed 2018).

Conversely, opaque goals may prompt misunderstanding and the potential for harms. For instance, when users of an AI system are unclear about what type of agent they are dealing with—human, artificial, or a hybrid combination of both—they may wrongly assume that the tacit norms of human-to-human social interaction are upheld (e.g., not recording every detail of a conversation) (Kerr 2003). As ever, the social context in which an AI4SG application takes place impacts the extent to which AI systems should be transparent in their operations. Because transparency is the default but not absolute position, there may be valid reasons for designers to obviate informing users of the software’s goals. For example, the scientific value of a project or the health and safety conditions of a public space may justify *temporarily* opaque goals. Consider a study that deceived students into believing that they were interacting with a human course-assistant that was in fact, over time, realised to be a bot (Eicher et al. 2017). The bot’s deception, as the authors argue, was for playing the “imitation game” without causing the students to choose simpler and less human-like natural-language queries based on preconceptions of AI capabilities. In such cases, the choice between opacity and transparency may be informed by preexisting notions of informed consent for human-subject experiments embedded in the Nuremberg Code, the Declaration of Helsinki, and the Belmont Report (Nijhawan et al. 2013).

More broadly, the ability to avoid the use of an AI system becomes more likely when AI software reveals its endogenous goals, like classifying data about a person. For example, AI software could inform staff in a hospital ward that it has the goal of classifying their hygiene levels (Haque et al. 2017). In this case, the staff may decide to avoid such classifications if there are reasonable alternative actions that they can take. In other cases, revealing a goal makes it less likely to be fulfilled.

Making transparent the goals and motivations of AI4SG developers themselves is an essential factor to the success of any project, but one that may contrast the very purpose of the system. This is why it is crucial to assess, at a design stage, what level of transparency (i.e. how much transparency, of what kind, for whom, and about what?) the project will embrace given its overall goal and the context of implementation. Taken together with the need for receiver-conceptualised explanation, this consideration yields the following set of best practices:

(4) AI4SG designers should choose a Level of Abstraction for AI explanation that fulfils the desired explanatory purpose and is appropriate to the system and the receivers; then deploy arguments that are rationally and suitably persuasive for the receivers to deliver the explanation; and ensure that the goal (the system’s purpose) for which an AI4SG system is developed and deployed is knowable to receivers of its outputs by default.

### Privacy Protection and Data Subject Consent

Of our seven factors, privacy has perhaps the most voluminous literature. This should not be a surprise, since privacy is considered to be an essential condition for safety, human dignity, and social cohesion, among other things (Solove 2008), and because earlier waves of digital technology have already had a major impact on privacy (Nissenbaum 2009). People’s safety may be compromised when a malicious actor or state gain control over individuals via privacy infringements (Taddeo 2015; Lynskey 2015). Respect for privacy is also a necessary condition of human dignity, since we can view personal information as constituting an individual, and deprivatising records without consent is likely to constitute a violation of human dignity (Floridi 2016). The conception of individual privacy as a fundamental right underlies recent legislative action in, for example, Europe (through its General Data Protection Regulation) and Japan (through its Act on Protection of Personal Information), as well as judicial decisions in jurisdictions such as India (Mohanty and Bhatia 2017). Privacy supports people in deviating from social norms without causing offense, and communities in maintaining their social structures, so privacy also undergirds social cohesion.

In the case of AI4SG, it is particularly important to emphasise the relevance of users’ consent to the use of personal data. Tensions may arise between different thresholds of consent (Price and Cohen 2019). The tension is often at its most fraught in “life-or-death” situations such national emergencies and pandemics. Consider the outbreak of Ebola in West Africa in 2014, which posed a complex ethical dilemma (*The *Economist 2014). In this case, the rapid release and analysis of call-data records from cell phone users in the region may have allowed epidemiologists to track the spread of the deadly disease. However, the release of the data was held up over valid concerns around users’ privacy, as well as the value of the data to industrial competitors.

In circumstances where haste is not so crucial, it is possible to obtain a subject’s consent for—and before—the data being used. The level or type of consent sought can vary with the context. In healthcare, one may adopt an assumed consent threshold, whereby reporting a medical issue to a doctor constitutes assumed consent on the part of a patient. In other circumstances, an informed consent threshold will be more appropriate. Yet, since informed consent requires researchers to obtain a patient’s specific consent before using their data for a non-consented purpose, practitioners may choose an explicit consent threshold to general data processing, i.e., for any medical usage. This threshold does not require informing the patient about all of the possible ways that researchers may use their data (Etzioni 1999). Another alternative is the evolving notion of “dynamic consent”, whereby individuals can monitor and adjust their privacy preferences on a granular level (Kaye et al. 2015).

In other cases, informed consent may be waived altogether. This was the case with the recent creation of machine learning software to predict the prognosis of ovarian cancer sufferers by drawing upon retrospective analysis of anonymised images (Lu et al. 2019). The use of patient health data in the development of AI solutions without patients’ consent has also attracted the attention of data protection regulators. In 2017, the UK’s Information Commissioner ruled that the Royal Free NHS Foundation Trust violated the Data Protection Act when it provided patient details to Google DeepMind, for the purposes of training an AI system to diagnose acute kidney injury (Burgess 2017). The Commissioner noted as a “shortcoming” that “patients were not adequately informed that their data would be used as part of the test” (“Royal Free—Google DeepMind Trial Failed to Comply with Data Protection Law” 2017).

Striking a balance between respecting patient privacy and creating effective AI4SG is still possible, however. This was the challenge faced by the researchers in Haque et al. (2017), who wanted to create a system for tracking compliance with rules around hand hygiene in hospitals, to prevent the spread of infections. Despite the clear technical advantages of taking a computer vision-based approach to the problem, the use of video recording runs up against privacy regulations constraining it. Even in cases where video recording is allowed, access to the recordings (in order to train an algorithm) is often strict. Instead, the researchers resorted to “depth images”, which de-identify subjects, preserving their privacy. While this design choice meant “losing important visual appearance cues in the process”, it satisfied privacy rules, and the researchers’ non-intrusive system still managed to outperform existing solutions.

Finally, consent in the online space is also problematic; users often lack the choice and are presented with a ‘take it or leave it’ option when accessing online services (Nissenbaum 2011; Taddeo and Floridi 2015). The relative lack of protection or consent for the second-hand use of personal data that is publicly shared online enables the development of ethically problematic AI software. For example, a recent paper used publicly available images of faces uploaded to a dating website as a way to train AI software to detect someone’s sexuality based on a small number of photos (Wang and Kosinski 2018). While the study received ethics committee approval, it raises further questions around consent, since it is implausible that the users of the dating website could or necessarily would have consented to the use of their data for this particular purpose.

Privacy is not a novel problem, but the centrality of personal data to many AI (and AI4SG) applications heightens its ethical significance and creates issues around consent (Taddeo and Floridi 2018a). From this we can derive the following best practice.

(5) AI4SG designers should respect the threshold of consent established for the processing of datasets of personal data.

### Situational fairness

AI developers typically rely on data, which may be biased in ways that are socially significant. This bias may carry across to the algorithmic decision-making that underpins many AI systems, in ways that are unfair to the subjects of the decision-making process (Caliskan, Bryson, and Narayanan 2017) and, thus, may breach the principle of justice. These decisions may be based on factors of ethical importance (e.g., ethnic, gender, or religious grounds) and irrelevant to the decision-making at hand, or they may be relevant but legally protected as a nondiscriminatory characteristic (Friedman and Nissenbaum 1996). Moreover, AI-driven decisions may be amalgamated from factors that are not of obvious ethical importance, and yet collectively constitute unfairly biased decision-making (Pedreshi et al. 2008; Floridi 2012).

AI4SG initiatives relying on biased data may propagate this bias through a vicious cycle (Yang et al. 2018). Such a cycle would begin with a biased dataset informing a first phase of AI decision-making, resulting in discriminatory actions, leading to the collection and use of biased data in turn. Consider the use of AI to predict preterm birth in the United States, where the health outcomes of pregnant women have long been affected by their ethnicity. Longstanding bias against African-American women seeking treatment, owing to harmful historical stereotypes, contributes to a maternal morbidity rate that is over three times higher than that of white women (CDC 2020). Here, AI may offer great potential to reduce this stark racial divide, but only if the same historical discrimination is not replicated in AI systems (Banjo 2018). Or consider the use of predictive policing software. Developers may train predictive policing software on policing data that contains deeply ingrained prejudices. When discrimination affects arrest rates, it becomes embedded in prosecution data (Lum and Isaac 2016). Such biases may cause discriminatory decisions (e.g., warnings or arrests) that feed back into the increasingly biased datasets (Crawford 2016), thereby completing a vicious cycle.

These examples involve the use of AI to improve outcomes in domains where data were already collected. Yet, in many other contexts, AI4SG projects (or indeed similar initiatives) are, in effect, making citizens “visible” in ways that they previously were not, including in global South contexts (Taylor and Broeders 2015). This increased visibility stresses the importance of protecting against the potential amplification of harmful bias by AI technologies.

Clearly, designers must sanitise the datasets used to train AI. However, there is equally a risk of applying too strong a disinfectant, so to speak, by removing important contextual nuances which could improve ethical decision-making. So, designers must also ensure that AI decision-making maintains sensitivity to factors that are important for inclusiveness. For instance, we should ensure that a word processor interacts identically with a human user regardless of that user’s gender and ethnicity, but also expect that it may operate in a non-equal and yet equitable way by aiding people with visual impairments.

Such expectations are not always met in the context of AI-driven reasoning. Compared to the word processor, AI makes possible a far wider range of decision-making and interaction modalities, many of which are driven by potentially biased data. Training datasets may contain natural language that carries unfair associations between genders and words which, in turn, carry normative power (Caliskan et al. 2017). In other contexts and use cases, an equitable approach may *require* differences in communication, based on factors such as gender. Consider the case of the virtual teaching assistant which *failed* to discriminate sufficiently well between men and women in its responses to being told that a user was expecting a baby, congratulating the men and ignoring the women (Eicher et al. 2017). A BBC News investigation highlighted an even more egregious example: a mental health chatbot deemed suitable for use by children was unable to understand a child explicitly reporting underage sexual abuse (White 2018). As these cases make clear, the use of AI in human–computer interactions, such as chatbots, requires the correct understanding of both the salient groups to which a user belongs and the characteristics they embody when they interact with the software.

Respecting situational fairness is essential for the successful implementation of AI4SG. To achieve it, AI4SG projects need to remove factors (and their proxies) that are of ethical importance but irrelevant to an outcome, and include the same factors when these are required, whether for the sake of inclusiveness, safety, or other ethical considerations. The problem of historical biases affecting future decision-making is an old one. What is new is the potential that these biases will be embedded in, strengthened, and perpetuated anew by erroneous reinforcement learning mechanisms. This risk is especially pronounced when considered alongside the risk of opacity in AI decision-making systems and their outcomes. We will return to this topic in the next section.

From our identification of situational fairness as an essential factor, we can yield the following best practice:

(6) AI4SG designers should remove from relevant datasets variables and proxies that are irrelevant to an outcome, except when their inclusion supports inclusivity, safety, or other ethical imperatives.

### Human-Friendly Semanticisation

AI4SG must allow humans to curate and foster their “semantic capital”, that is,

any content that can enhance someone’s power to give meaning to and make sense of (*semanticise*) something (Floridi et al. 2018).

This is crucial to maintain and foster human autonomy. With AI, we may often have the technical capacity to automate meaning- and sense-creation (semanticisation), but mistrust or unfairness may also arise if we do so carelessly. Two problems emerge. The first problem is that AI software may define semanticisation in a way that diverges from our own choices. This is the case if a procedure arbitrarily defines meanings (e.g., based on a coin toss). The same problem may arise if AI software support some kind of semanticisation based on preexisting uses. For example, researchers have developed an application that *predicts the legal meaning* of ‘violation’ based on past cases (Al-Abdulkarim et al. 2015). If one used the software to *define* the meaning of ‘violation’,^9^ then one would end up limiting the role of judges and justices. They would no longer be able to semanticise (refine and re-define the meaning, and the possibility of making sense of) “violation”, when they interpret the law. This is a problem, because past usage does not always predict how we would semanticise the same concepts or phenomena in the future.

The second problem is that, in a social setting, it would be impractical for AI software to define all meanings and senses. Some semanticisation is subjective, because who or what is involved in the semanticisation is also partly constitutive of the process and its outcome. For example, only legally empowered agents can define the legal meaning of ‘violation’. Likewise, the meaning and sense of affective symbols, such as facial expressions, also depends on the type of agent showing a given expression. Affective AI can detect an emotion (Martínez-Miranda and Aldea 2005), an artificial agent may state accurately that a human *appears sad*, but cannot change the meaning of sadness.

The solution to these two problems rest on distinguishing between tasks that should and should not be delegated to an artificial system. AI should be deployed to *facilitate* human-friendly semantisation, but not to provide it itself. This is true, for example, when considering patients with Alzheimer’s disease. Research into carer-patient relations highlights three points (Burns and Rabins 2000). First, carers play a critical, but burdensome, role in reminding patients of the activities in which they participate, e.g., taking medication. Second, carers also play a critical role in providing patients with meaningful interaction. And third, when carers remind patients to take their medication, the patient-carer relation may become weaker by annoying the patient, with the carer losing some capacity to provide empathy and meaningful support. Consequently, researchers have developed AI software that balances reminding the patient against annoying the patient (Chu et al. 2012). The balance is learned and optimised using reinforcement learning. The researchers designed the system so that caregivers can spend most of their time providing empathic support and preserving a meaningful relationship with the patient. As this example shows, it is possible to use AI to sweep away formulaic tasks whilst sustaining human-friendly semanticisation.

Human-centric semanticisation, as an essential factor for AI4SG, underpins our final best practice:

(7) AI4SG designers should not hinder the ability for people to semanticise (that is, to give meaning to, and make sense of) something.

## Conclusion: Balancing Factors for AI for Social Good

The seven factors analysed in the previous pages are summarised in Table 1, together with the corresponding best practices, and the five principle(s) of AI ethics identified in (Floridi and Cowls 2019) to which each factor is most closely identified. To reiterate, the principle of beneficence is assumed as a precondition for an AI4SG, so the factors relate to one or more of the other four principles: nonmaleficence, autonomy, justice and explicability.

**Table 1:** Summary of seven factors supporting AI4SG and the corresponding best practices

Factors	Corresponding best practices	Corresponding ethical principle
Falsifiability and incremental deployment	Identify falsifiable requirements and test them in incremental steps from the lab to the “outside world”	Nonmaleficence
Safeguards against the manipulation of predictors	Adopt safeguards which (i) ensure that non-causal indicators do not inappropriately skew interventions, and (ii) limit, when appropriate, knowledge of how inputs affect outputs from AI4SG systems, to prevent manipulation	Nonmaleficence
Receiver-contextualised intervention	Build decision-making systems in consultation with users interacting with and impacted by these systems; with understanding of users’ characteristics, the methods of coordination, the purposes and effects of an intervention; and with respect for users’ right to ignore or modify interventions	Autonomy
Receiver-contextualised explanation and transparent purposes	Choose a Level of Abstraction for AI explanation that fulfils the desired explanatory purpose and is appropriate to the system and the receivers; then deploy arguments that are rationally and suitably persuasive for the receiver to deliver the explanation; and ensure that the goal (the system’s purpose) for which an AI4SG system is developed and deployed is knowable to receivers of its outputs by default	Explicability
Privacy protection and data subject consent	Respect the threshold of consent established for the processing of datasets of personal data	Nonmaleficence; autonomy
Situational fairness	Remove from relevant datasets variables and proxies that are irrelevant to an outcome, except when their inclusion supports inclusivity, safety, or other ethical imperatives	Justice
Human-friendly semanticisation	Do not hinder the ability for people to semanticise (that is, to give meaning to, and make sense of) something	Autonomy

The seven factors suggest that creating successful AI4SG requires two kinds of balances to be struck: *intra* and *inter*.

On the one hand, each single factor in and of itself may require an intrinsic balance, for example, between the risk of over-intervening and the risk of under-intervening when devising contextual interventions; or between protection-by-obfuscation and protection-by-enumeration of salient differences between people, depending on the purposes and context of a system. On the other hand, balances are not just specific to a single factor; they are also systemic, because they must also be struck between multiple factors. Consider the tension between preventing malicious actors from understanding how to “game” the input data of AI prediction systems versus enabling humans to override genuinely flawed outcomes; or the tension between ensuring the effective disclosure of the reasons behind a decision without compromising the consensual anonymity of data subjects.

The overarching question facing the AI4SG community is, for each given case, whether one is morally obliged to, or obliged not to, design, develop, and deploy a specific AI4SG project. This article does not seek to answer such a question in the abstract. Resolving the tensions that are likely to arise among and between factors is highly context-dependent, and the previous analysis is not meant to cover all potential contexts, not least because this would be inconsistent with the argument for falsifiable hypothesis testing and incremental deployment supported in this article; nor would a checklist of purely technical “dos and don’ts” suffice. Rather, our analysis has yielded a set of essential factors that need to be considered, interpreted and evaluated contextually when one is designing, developing, and deploying a specific AI4SG project. The future of AI4SG will likely provide more opportunities to enrich such a set of essential factors. AI itself may help to manage its own life cycle by providing, in a meta-reflective way, tools to evaluate how best to strike the individual and systemic balances indicated above.

The most pertinent questions to arise from the factors described in this article are likely to concern this challenge of balancing the competing needs and claims that the factors and corresponding best practices introduce. This concerns what it is that *legitimates* decision-making with and about AI. While we leave this concern primarily to future research, we offer some remarks on it in closing. Questions such as this are inevitably intertwined with wider ethical and political challenges regarding who has the power or “standing” to participate in this process of evaluation, as well as how multiple preferences are measured and aggregated, as Baum’s trichotomic framework outlines (Baum 2017). If we assume that the challenge of balancing factors ought to be at least somewhat participatory in nature, Prasad’s (2018) overview of relevant social choice theorems identifies several background conditions to support efficacious group decision-making. As these analyses suggest, the incorporation of multiple perspectives into the design of AI decision-making systems is likely to be an ethically important step both for AI in general, and AI4SG in particular.

There is much work still to be done to ensure that AI4SG projects are designed in ways that not merely advance beneficial goals and address societal challenges, but that do so in socially preferable and sustainable ways. This article seeks to contribute to lay the ground for good practices and policies in this respect, as well as for further research on the ethical considerations that should undergird AI4SG projects, and hence *the* “AI4SG project” at large.

## Appendix: Representative AI4SG Examples

In the table below, we list the seven initiatives from our wider sample that are especially representative in terms of scope, variety, impact, and for their potentiality to evince the factors that should characterise the design of AI4SG projects. This includes the factor(s) that were identified as a result of our analysis of each project.

**Table:** 

	Name	References	Areas	Relevant factor(s)
A	Field optimization of the protection assistant for wildlife security	Fang et al. (2016)	Environmental sustainability	(1), (3)
B	Identifying students at risk of adverse academic outcomes	Lakkaraju et al. (2015)	Education	(4)
C	Health information for homeless youth to reduce the spread of HIV	Yadav et al. (2016a,b), Yadav et al. (2018)	Poverty, public welfare, public health	(4)
D	Interactive activity recognition and prompting to assist people with cognitive disabilities	Chu et al. (2012)	Disability, public health	(3), (4), (7)
E	Virtual teaching assistant experiment	Eicher et al. (2017)	Education	4), 6)
F	Detecting evolutionary financial statement fraud	Zhou and Kapoor (2011)	Finance, crime	(2)
G	Tracking and monitoring hand hygience compliance	Haque et al. (2017)	Health	(5)

## Footnotes

1 While it is beyond present scope to adjudicate this for any particular case, it is important to acknowledge at the outset that in practice there is likely to be considerable disagreement and contention regarding what would constitute a socially good outcome.

2 This should not be taken as necessitating a utilitarian calculation: the beneficial impact of a given project may be “offset” by the violation of some categorical imperative. Therefore even if an AI4SG project would do “more good than harm”, the harm may be ethically intolerable. In such a hypothetical case, one would not be morally obliged to develop and deploy the project in question.

3 As noted in the introduction, we cannot hope to document every single ethical consideration for a social good project, so even the least novel factors here are those that take on new relevance in the context of AI.

4 It is of course likely that in practice, an assessment of the safety of an AI system must also take into account wider societal values and cultural beliefs, for example, which may necessitate different trade-offs between the requirements of critical requirements like safety and other, potentially competing norms and expectations.

5 While, for the sake of simplicity, our focus is on *minimising* the spread of information used to predict an outcome, we do not intend to foreclose on the suggestion, offered in Prasad (2018), that in some cases a fairer approach may be to *maximise* the available information and hence “democratise” the ability to manipulate predictors.

6 For a discussion of the use of artificial intelligence in criminal acts more generally, see King et al. 2019.

7 The four remaining dimensions proposed by MacFarlane—the source of the interruption, the method of expression, the channel of conveyance and the human activity changed by the interruption—are not relevant for purpose of this article.

8 Note that the significance of involving domain experts in the process was not merely to improve their experience as decision recipients, but also for their unparalleled knowledge of the domain that the researchers drew upon in the system design, helping to provide the researchers with what Pagallo (2015) calls “preventive understanding” of the field.

9 There is no suggestion that this is the intended use.
